# Development of a Virtual Reality Program for Internationally Standardized Non-Face-to-Face Nursing Practicum Education: Design and Validation of a Sensor-Integrated XR System

**DOI:** 10.3390/s26061843

**Published:** 2026-03-14

**Authors:** Ji Won Oak

**Affiliations:** Department of Nursing, Tongmyong University, 428, Sinseon-ro, Nam-gu, Busan 48520, Republic of Korea; jiwonoak@gmail.com

**Keywords:** extended reality (XR), automated performance assessment, motion sensing, sensor-integrated simulation, performance measurement in XR

## Abstract

**Highlights:**

**What are the main findings?**

- A controller-free, sensor-integrated XR nursing practicum system enabled precise capture and quantification of fine motor and procedural performance.
- Automated XR-based assessment demonstrated discriminatory power comparable to instructor-based evaluation and was technically validated through accredited V&V testing.

**What are the implications of the main findings?**

- Precision sensing transforms XR from an immersive training tool into a reproducible, measurement-oriented assessment system for nursing skills education.
- The proposed framework supports data-driven standardization of non-face-to-face nursing practicum education across institutions and contexts.

**Abstract:**

Extended reality (XR) has increasingly been applied to nursing practicum education; however, most systems rely on controller-based interfaces that limit precise capture of continuous fine motor performance and objective assessment. This study developed and validated a sensor-integrated, controller-free XR nursing practicum system (Smart Nursing v1.0) grounded in continuous precision sensing. Based on internationally standardized intravenous injection protocols, the system integrated optical hand tracking and speech recognition to quantify hand kinematics, spatial accuracy, procedural sequencing, and verbal compliance. A three-phase validation framework was implemented. Internal technical verification confirmed stable real-time performance (≥60 FPS) and consistent action recognition. In a user-based study involving 63 undergraduate nursing students, XR-based automated scores demonstrated high agreement with expert instructor ratings (ICC = 0.932, 95% CI = 0.91–0.96, *p* < 0.001). XR baseline scores significantly predicted post-training performance (β = 0.632, *p* < 0.001) and showed significant incremental validity beyond instructor pre-training scores (ΔR^2^ = 0.186, *p* < 0.001). Independent verification confirmed high recognition accuracy (100%) and system stability. These findings indicate that precision sensing enables XR environments to function as reliable performance measurement systems, supporting standardized non-face-to-face nursing practicum education.

## 1. Introduction

Extended Reality (XR), encompassing Virtual Reality (VR), Augmented Reality (AR), and Mixed Reality (MR), refers to technologies that reconstruct real-world contexts within immersive digital environments, thereby enabling enhanced learner engagement and repeated practice. In recent years, XR has attracted increasing attention in medical and healthcare education as an alternative educational modality capable of mitigating the constraints and risks associated with real clinical environments. Its applicability has expanded particularly in domains that require high procedural accuracy and performance consistency, such as clinical skills training [1,2,3].

In nursing education, practicum-based training constitutes a core component alongside theoretical instruction, as it enables learners to acquire and integrate psychomotor skills and clinical decision-making competencies essential for real clinical practice. However, conventional face-to-face nursing practicum education is subject to persistent structural limitations, including restricted access to clinical placements, disparities in educational infrastructure and instructor expertise, and inconsistencies in assessment criteria across institutions and countries. These limitations contribute to substantial variability in learning outcomes [4]. Such challenges became more pronounced following the rapid expansion of non-face-to-face education after the COVID-19 pandemic, resulting in increased demand for internationally applicable and standardized nursing practicum education systems [5].

Previous studies on XR-based nursing and healthcare education have consistently reported positive effects of virtual simulation on learners’ self-efficacy, confidence in clinical skill performance, knowledge retention, and readiness for clinical practice [2,6,7]. In particular, core nursing skills such as intravenous injection and catheter insertion involve direct patient safety risks, underscoring the educational value of XR-based repetitive and risk-free training environments [8]. In this context, XR has been regarded as a promising approach for implementing standardized non-face-to-face practicum education, as it enables the delivery of identical educational scenarios independent of physical location or training resource constraints.

Nevertheless, XR-based nursing practicum systems continue to exhibit critical limitations. While some studies suggest that high levels of visual immersion alone may be sufficient to achieve meaningful learning outcomes [9], others argue that effective transfer to real clinical performance requires more than visual realism, emphasizing the importance of functional fidelity [10]. Many existing XR systems rely on generic handheld controllers or simplified gesture-based inputs, which are insufficient for capturing essential characteristics of clinical skill execution, including hand position and orientation, fine motor continuity, and procedural sequencing. As a result, such systems have been criticized for their limited capacity to represent authentic clinical performance [9,10].

Recent research has increasingly converged on the view that these limitations stem not from insufficient visual realism, but from the absence of sensor-based input structures capable of precisely detecting and quantifying the multidimensional characteristics of clinical skill performance [11,12]. Nursing skills do not consist of isolated actions; rather, they comprise complex processes that integrate fine hand movements, spatial accuracy, temporal coordination, procedural compliance, and context-dependent verbal performance. From the perspective of international standardization, sensor-based XR systems are therefore regarded as essential, as they enable objective recording and analysis of learner performance data, thereby supporting consistency in educational content and assessment criteria across diverse institutional and cultural contexts [4,11].

Moreover, sensor-based XR systems facilitate the quantitative representation of performance data, enabling meaningful comparisons across learners, institutions, and countries and reducing variability in educational quality. This data-driven approach provides a technological foundation for establishing internationally agreed-upon educational standards by enhancing the reproducibility and comparability of performance outcomes [5,12]. Recent XR studies have further demonstrated that the integration of gesture recognition, motion tracking, speech recognition, and sensor fusion technologies significantly improves the accuracy and objectivity of performance assessment within XR environments [12,13,14].

Accordingly, this study aims to develop a sensor-integrated, controller-free XR-based nursing practicum system designed to support internationally standardized non-face-to-face nursing education and to validate the technical feasibility and methodological applicability of sensor-derived objective performance metrics.

The proposed system will employ continuous sensing mechanisms to capture and quantify learners’ motor and verbal performance, thereby enabling an objective and structured representation of nursing skill execution.

Through this approach, the study seeks to (1) enhance functional fidelity and objectivity in XR-based nursing practicum education and (2) provide methodological and technical evidence to support the future development of standardized XR-based nursing education and assessment frameworks.

## 2. Materials and Methods

### 2.1. Study Design Overview

This study was designed as a three-phase validation study to examine the technical robustness and measurement validity of a sensor-based XR nursing skills assessment system, Smart Nursing v1.0 (Gridatech Inc., Busan, Republic of Korea).

Phase 1: System Development and Internal Technical Verification

A sensor-integrated XR platform was developed based on internationally standardized nursing skills protocols. The system underwent internal technical verification to ensure functional stability, protocol fidelity, and measurement consistency.

Phase 2: User-Based Criterion Validity Assessment

The automated measurement function was implemented in authentic undergraduate nursing education settings. Criterion-related validity was examined through comparative analysis with expert instructor evaluations.

Phase 3: Independent Engineering Verification and Validation (V&V)

Engineering reliability, system stability, and compliance with technical standards were independently verified through formal V&V procedures conducted by an accredited testing institution.

This study aims to establish the technical soundness and measurement validity of an XR-based automated assessment system, rather than to evaluate its comparative educational effectiveness.

### 2.2. Phase 1: System Development and Internal Technical Validation

#### 2.2.1. System Design and Architecture

The intravenous injection training XR program developed in this study was grounded in a measurement-oriented XR framework, conceptualizing sensors not as simple interaction devices but as continuous measurement instruments. The system was designed to quantitatively capture and analyze fine hand movements and procedural sequences occurring during nursing skill performance.

The hardware setup consisted of a Meta Quest 2 head-mounted display (HMD) equipped with a front-mounted Leap Motion optical hand-tracking sensor. The Leap Motion sensor collected 3D joint keypoints (position and orientation data) at an effective sampling rate of approximately 30–60 Hz, transmitting data to the XR environment in real time through a TCP/IP-based communication framework.

Collected sensor data underwent timestamp-based synchronization with procedural stages in the XR simulation environment. This synchronization enabled reconstruction of joint kinematics, spatial coordinates, movement trajectories, and motion durations with temporal resolution.

Unlike conventional controller-based XR systems, the present system processed hand tracking and voice recognition data as continuous sensor streams rather than discrete event-triggered inputs. This architecture enabled quantification of spatial accuracy, temporal consistency, and procedural sequencing during intravenous injection procedures.

#### 2.2.2. Behavior Recognition Logic Based on International Standards

The behavior recognition model was designed through comparative analysis of domestic and international nursing skill guidelines. The following standards were analyzed:

- Core Basic Nursing Skills (Version 4.1)
- United States Intravenous Injection Nursing Guidelines
- Australian Clinical Nursing Skills Guidelines

Common procedural elements emphasized across the three guidelines were identified, including patient identification, aseptic technique compliance, application of the 5 Rights principle, appropriate venipuncture site selection, and insertion and fixation procedures.

Each procedural element was operationalized into quantifiable behavioral indicators measurable within the XR environment. Primary indicators included:

- Hand joint angle variations
- Distance between hands and instruments
- Spatial accuracy within the target area
- Procedural sequence consistency
- Minimum dwell time and inter-stage temporal continuity

Behavior recognition was implemented using sliding temporal windows of 0.5–1.5 s. A procedural stage was classified as completed when the following three conditions were simultaneously satisfied:

- Kinematic criteria fulfillment (motion magnitude and directionality)
- Temporal consistency fulfillment (minimum duration and sequence matching)
- Protocol congruence fulfillment (contextual validity of procedural stage)

Threshold values were established through expert review and pilot log analysis. Range-based tolerance intervals were applied rather than fixed absolute thresholds to accommodate inter-user variability.

#### 2.2.3. Automated Assessment Structure Design

Automated assessment was designed based on a 50-item checklist with expert-validated content validity. Each item was mapped to sensor-based behavior recognition outcomes for scoring.

Item scores were calculated through a four-step process:

- Procedural stage determination
- Performance accuracy indicator calculation
- Temporal and sequence consistency verification
- Score assignment upon fulfillment of predefined criteria

The final total score was computed as the sum of individual item scores, maintaining structural equivalence with conventional instructor-based evaluation frameworks.

#### 2.2.4. Internal Technical Validation

Prior to user-based validation, internal technical verification was conducted to ensure functional stability and system integrity under real-time XR conditions. The verification process focused on the following components:

- Hand keypoint recognition stability: Continuous tracking accuracy and temporal consistency of three-dimensional hand joint data were evaluated under repeated task execution.
- Behavior recognition algorithm functionality: The rule-based action recognition logic was tested using standardized procedural sequences to confirm correct step detection and logical consistency.
- Real-time processing performance: System responsiveness and frame rate stability were monitored to ensure maintenance of ≥60 frames per second (FPS) during active interaction.
- Data integrity verification: Logging records were examined to detect frame drops, data packet loss, or latency anomalies during extended runtime sessions.

Each component underwent repeated verification testing across multiple sessions. The system demonstrated stable operation without critical tracking loss, processing interruption, or performance degradation under real-time XR conditions.

#### 2.2.5. Measurement Agreement Validation

To examine the measurement agreement of the automated scoring structure, the absolute agreement between XR-based automated scores and expert (instructor) ratings was evaluated using the intraclass correlation coefficient (ICC).

Both XR-based automated scoring and instructor ratings were derived from identical standardized performance cases to ensure comparability.

The ICC was calculated using a two-way random-effects model with absolute agreement and single measures. This analysis was conducted to determine the extent to which the automated assessment system produced scores consistent with expert-based evaluations under controlled and standardized performance conditions.

### 2.3. Phase 2: User-Based Criterion Validity Assessment

#### 2.3.1. Participants and Ethical Considerations

This study was conducted at a university in South Korea. Participants consisted of 63 second-year nursing students who had not yet completed formal intravenous injection practicum training but had engaged in VR-based self-practice programs for at least three months as part of their regular curriculum.

Sample size was calculated using G*Power 3.1.9.7. Based on a medium effect size (d = 0.50) as suggested by Cohen [15], an alpha level of 0.05, statistical power of 0.95, and a two-tailed test, the minimum required sample size was 54. A total of 63 students voluntarily participated in the study.

The study was approved by the Institutional Review Board (TUIRB-2021-007). All participants received detailed explanations regarding the study purpose and procedures and provided written informed consent prior to participation.

Participant characteristics are summarized in Table 1. The sample consisted of 82.5% females (*n* = 52). Most participants reported medium academic performance (55.6%, *n* = 35), and 74.6% reported medium or high levels of major satisfaction.

#### 2.3.2. Experimental Procedure

Participants completed the experiment in the following sequence:

- Traditional instructor-led training, consisting of a lecture, demonstration, and a 30 min self-practice session.
- Instructor- evaluation conducted using the standardized 50-item checklist. The instructor performed scoring only, and no feedback was provided at this stage.
- XR-based baseline automated assessment, conducted in evaluation mode without automated feedback. This assessment captured participants’ baseline performance using the sensor-based measurement logic developed in Phase 1.
- XR free practice session (30 min), conducted in practice mode with automated stage-specific feedback.
- Instructor post-evaluation using the identical 50-item checklist. Scoring was completed prior to providing performance feedback.

This sequence ensured that both the instructor pre-assessment and the XR baseline assessment were obtained prior to the feedback-based XR practice intervention.

#### 2.3.3. Measurement Instruments

(1) Instructor Evaluation Tool

Instructor evaluation was conducted using a 50-item checklist developed by the researcher and two nursing professors. Content validity was verified by five nursing experts (S-CVI/Ave = 0.98). Items were rated on a 5-point Likert scale, yielding a maximum total score of 250 points.

The instrument comprised four subscales:

- Nursing Knowledge (10 items)
- Acquisition (5 items)
- Clinical Skills (30 items)
- Learning Satisfaction (5 items)

Higher scores indicated better performance.

(2) XR-Based Automated Assessment

The XR automated assessment system utilized the identical 50-item checklist structure. Each item was automatically scored using sensor-based behavior recognition algorithms converted into quantitative performance indicators.

(3) User Evaluation Tool (Usability and Technical Stability Assessment)

User evaluation was conducted using a structured questionnaire co-developed by the researcher and two engineering professors, with verified content validity. The usability-related domains were conceptually structured in alignment with the core dimensions defined in ISO 9241-11 [16] (effectiveness, efficiency, and satisfaction), thereby supporting methodological transparency and conceptual reproducibility.

The questionnaire consisted of five domains (three items each):

- Ease of Use
- Interface Intuitiveness
- System Stability
- Immersion
- Overall Usability

Each item was rated on a 5-point Likert scale (score range per domain: 3–15), with higher scores indicating more favorable evaluations.

Although a fully standardized instrument such as the System Usability Scale (SUS) was not formally administered, the questionnaire design was informed by internationally recognized usability principles to enhance cross-study comparability.

A separate technical stability checklist was used to assess the occurrence of system errors, latency, tracking loss, and screen freezing during XR operation.

#### 2.3.4. Statistical Analysis

All statistical analyses were performed using SPSS version 28.0 (IBM Corp., Armonk, NY, USA).

(1) Learning Effect

To examine the educational effect of XR-based training, differences between instructor-rated pre- and post-assessment scores were analyzed using paired *t*-tests.

To examine whether learning gains differed according to learner characteristics, change scores were calculated as:

Δ = Post Instructor − Pre Instructor

Independent *t*-tests were used for gender comparisons, and one-way ANOVA was used for academic achievement and major satisfaction.

(2) Predictive and Incremental Validity of XR Assessment

To examine the predictive validity of the XR-based automated assessment, linear regression analyses were conducted to determine whether baseline XR assessment scores predicted instructor-rated post-training performance.

Additionally, hierarchical regression analysis was performed to examine incremental validity:

- Step 1: Instructor post-score was regressed on instructor pre-score.
- Step 2: XR baseline score was added to the model.

The change in explained variance (ΔR^2^) was examined to determine whether XR baseline scores provided additional explanatory power beyond baseline instructor-rated performance.

(3) User Evaluation Analysis

Descriptive statistics, including means and standard deviations, were calculated for each usability domain (ease of use, interface intuitiveness, system stability, immersion, and overall usability).

(4) Technical Stability Analysis

The frequency and percentage of system errors, delays, tracking loss, and screen freezing events occurring during XR use were calculated and reported descriptively.

### 2.4. Phase 3: Independent Engineering Verification and Validation (V&V)

#### 2.4.1. Verification and Validation Procedure

Following user-based validation, independent engineering Verification and Validation (V&V) procedures were conducted on the finalized Smart Nursing v1.0 system by an accredited testing institution.

The purpose of this verification was to objectively confirm the system’s technical reliability, measurement accuracy, and real-time processing stability. The evaluation was performed by an external institution independent of the development team to ensure procedural objectivity and technical impartiality.

#### 2.4.2. Verification Components

The verification components were structured as follows:

- Hand Keypoint Recognition Accuracy: Three-dimensional hand joint coordinate tracking accuracy was evaluated by comparing system outputs with reference (ground-truth) data.
- XR-Based Behavior Recognition Accuracy: Standardized procedural performance scenarios were applied to assess the accuracy of stage-wise behavior recognition outcomes.
- Real-Time Processing Performance (FPS Stability): System performance was evaluated by determining whether the frame rate (FPS) was stably maintained at or above the predefined criterion (≥60 FPS) during runtime.

Each verification component was evaluated according to predefined pass criteria.

#### 2.4.3. Evaluation Criteria

The evaluation criteria were defined as follows:

- Hand keypoint recognition accuracy: Achievement of 100% recognition accuracy relative to reference data
- Behavior recognition accuracy: Consistency with standardized procedural stages
- Processing performance: Maintenance of an average frame rate of ≥60 FPS

All verification components satisfied the predefined pass criteria.

## 3. Results

### 3.1. Implementation of the XR-Based Intravenous Injection Training Program and Measurement Agreement

**Figure 1:**
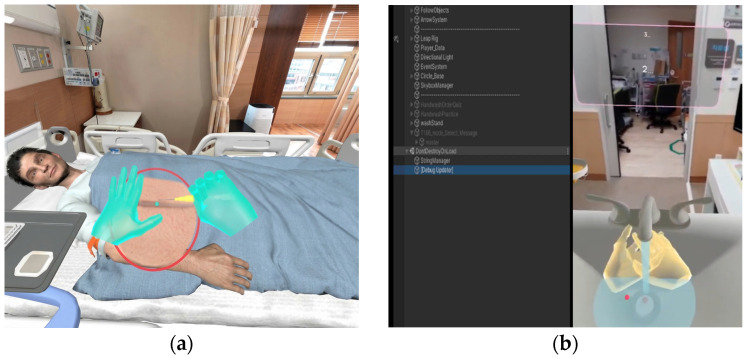
Sensor-integrated XR environment for intravenous nursing skills training. (**a**) XR-based intravenous injection scene showing real-time hand keypoint tracking and interaction with the patient avatar during the injection procedure. (**b**) XR hand hygiene training scene illustrating hand motion tracking and real-time visual guidance in a simulated clinical sink environment.

A sensor-integrated XR-based intravenous injection training system was successfully implemented based on a measurement-oriented architecture. The system enabled real-time acquisition and synchronization of three-dimensional hand joint keypoints with procedural stages in the XR environment. The virtual environment realistically simulated a clinical hospital room, hand hygiene station, patient avatar, and intravenous injection equipment, as illustrated in Figure 1. Major procedural stages—including hand hygiene, material preparation, venipuncture site approach, catheter insertion, and fixation—were fully integrated with the automated assessment algorithm. Internal technical validation confirmed stable operation without frame rate degradation or tracking loss under repeated execution conditions. To evaluate measurement agreement between XR-based automated scoring and expert instructor evaluation, the intraclass correlation coefficient (ICC) was calculated using a two-way random-effects model with absolute agreement for single measures (ICC [2, 1]). The analysis demonstrated a very high level of agreement (ICC = 0.932, 95% CI = 0.91–0.96, F(4, 20) = 15.23, *p* < 0.001), indicating strong consistency between automated and expert scoring.

### 3.2. Educational Effects of XR-Based Training

#### 3.2.1. Comparison of Educational Outcomes Before and After XR-Based Training (N = 63)

Instructor-rated pre- and post-assessment scores were compared using paired *t*-tests to examine the educational effects of the XR-based training, as shown in Table 2. Significant improvements were observed across all outcome domains.

Nursing knowledge scores significantly increased following XR-based training (t = 10.624, *p* < 0.001). Acquisition scores also demonstrated significant improvement (t = 11.815, *p* < 0.001). Clinical skills performance showed a substantial increase after training (t = 10.961, *p* < 0.001). Learning satisfaction similarly improved significantly (t = 8.370, *p* < 0.001).

#### 3.2.2. Differences in Learning Gains According to Learner Characteristics

To examine whether learning gains differed according to learner characteristics, change scores (Δ = post-instruction score − pre-instruction score) were analyzed (Table 3).

A statistically significant difference in learning gains was observed according to gender (t = −2.791, *p* = 0.007). In addition, learning gains differed significantly by academic achievement level, F(2, 60) = 4.435, *p* = 0.016, and by major satisfaction, F(2, 60) = 3.654, *p* = 0.032.

Post hoc comparisons using Tukey’s HSD indicated that students in the low academic achievement group demonstrated significantly greater learning gains than those in the high academic achievement group (*p* < 0.05). No significant differences were found between the medium and high groups or between the low and medium groups.

Similarly, Tukey’s post hoc analysis revealed that students with low major satisfaction showed significantly greater learning gains than those with high major satisfaction (*p* < 0.05), whereas differences between other group pairs were not statistically significant.

#### 3.2.3. Predictive and Incremental Validity of XR-Based Assessment

To examine the predictive validity of the XR-based automated assessment, simple linear regression analysis was conducted to determine whether baseline XR scores predicted instructor-rated post-training performance. Additionally, hierarchical regression analysis was performed to examine whether XR baseline scores provided incremental explanatory power beyond instructor pre-training performance.

The results are presented in Table 4.

Simple linear regression analysis indicated that XR baseline scores significantly predicted instructor-rated post-training performance (β = 0.632, *p* < 0.001), explaining 39.9% of the variance (R^2^ = 0.399).

In the hierarchical regression analysis, instructor pre-training scores significantly predicted post-training performance in Step 1 (β = 0.602, *p* < 0.001), accounting for 36.2% of the variance (R^2^ = 0.362).

When XR baseline scores were added in Step 2, the model’s explanatory power increased significantly (ΔR^2^ = 0.186, *p* < 0.001). Both instructor pre-training scores (β = 0.389, *p* < 0.001) and XR baseline scores (β = 0.452, *p* < 0.001) remained significant predictors. The final model explained 54.8% of the variance in post-training performance.

These findings demonstrate that the XR-based automated assessment not only predicts instructor-rated clinical performance but also provides significant incremental validity beyond baseline instructor evaluation.

### 3.3. Usability Evaluation Results of the XR Program

The usability evaluation results of the XR-based intravenous nursing training program are presented in Table 5. The overall usability score was 4.59 ± 0.42 on a 5-point scale. The mean scores for individual items were 4.52 ± 0.48 for ease of use, 4.61 ± 0.44 for interface intuitiveness, 4.58 ± 0.51 for system stability, and 4.67 ± 0.39 for immersion. Satisfaction rates for all items exceeded 90%.

### 3.4. Technical Stability Evaluation Results of the XR Program

The technical stability-related outcomes observed during XR program use are presented in Table 6. Among the 63 participants, no system errors or dropouts occurred. VR sickness and device malfunction were each reported by one participant (1.6%), and the overall completion rate was 100%.

### 3.5. Technical Validation Results from an Accredited Testing Institution

After completion of the user evaluation, verification and validation testing of the Smart Nursing v1.0 system was conducted by an accredited testing institution. The results are presented in Table 7.

The labeling syntax accuracy of the action recognition dataset was measured at 100%, satisfying the acceptance criterion of ≥ 99.5%. The keyword matching accuracy of the speech-to-text (STT) model based on text parsing averaged approximately 98.7%. Both user hand keypoint recognition accuracy and VR-based nursing action recognition accuracy were measured at 100%. The user hand keypoint detection speed was maintained at 60 FPS or higher (Table 7).

## 4. Discussion

This study aimed to develop and validate a sensor-integrated, controller-free XR-based nursing practicum system as a measurement-oriented training framework grounded in continuous precision sensing. Unlike prior XR-based nursing education research that has primarily emphasized learner satisfaction, immersion, or self-efficacy outcomes [2,6,7], the present study examined whether continuous sensor streams could be systematically structured into performance metrics comparable to expert evaluation. In doing so, XR is repositioned from an immersive instructional interface to a sensor-based performance measurement infrastructure.

### 4.1. Structural Agreement with Expert Evaluation

The high level of agreement observed between XR automated assessment scores and instructor ratings (ICC = 0.932) indicates that the proposed sensing architecture can reliably reproduce the structural logic of expert-based evaluation under standardized conditions. Learning analytics research has consistently emphasized that transforming raw sensor data into standardized performance indicators is a prerequisite for objective and scalable assessment [17]. The present findings operationalize this principle within a nursing practicum context.

Furthermore, AR/MR research identifies spatial registration accuracy and coordinate consistency as fundamental system properties rather than auxiliary features [18,19]. In medical simulation, the standardization and reliability of performance assessment are regarded as essential for expanding clinical training frameworks [20]. The strong structural agreement demonstrated in this study supports the feasibility of XR as a reproducible measurement environment rather than merely a visualization platform.

### 4.2. Incremental Validity and Continuous Signal-Based Assessment

A central contribution of this study lies in the incremental validity demonstrated by XR-derived baseline scores. After controlling for instructor pre-training performance, XR baseline scores explained additional variance in post-training performance (ΔR^2^ = 0.186). This indicates that sensor-based performance indicators capture procedural characteristics not fully represented in conventional checklist-based assessments.

Traditional clinical skill evaluations frequently rely on categorical or stage-based judgments [17], which may lack sensitivity to subtle motor deviations and temporal coordination differences. In contrast, continuous signal-based analysis has long been utilized in medical and biosensing domains for fine-grained pattern recognition and performance discrimination [21]. Recent advances in procedural skills assessment further advocate integrating motion-tracking and algorithmic analytics to enhance precision and objectivity [22,23]. By incorporating continuous hand joint tracking and procedural sequencing logic, the present study extends these approaches into XR-based nursing skills assessment and empirically demonstrates expanded discriminatory capacity.

### 4.3. Functional Fidelity and Perception–Action Coupling

Although significant improvements were observed across knowledge, acquisition, clinical skills, and learning satisfaction domains, the primary contribution of this work lies in structurally enhancing functional fidelity rather than merely demonstrating learning gains. Prior research has indicated that controller-based XR systems may inadequately represent spatial accuracy, procedural continuity, and fine motor execution [9,10]. The integration of continuous hand tracking and algorithm-based procedural recognition in the present system addresses these limitations.

Embodied interaction research suggests that tightly coupled perception–action loops facilitate motor learning and performance precision [24,25]. By grounding evaluation logic directly in real-time kinematic data, the proposed system operationalizes embodied interaction principles within a measurable and reproducible XR environment. This strengthens alignment between physical execution and digital assessment criteria.

### 4.4. Independent Engineering Validation and Reproducibility

Many XR education studies rely predominantly on subjective usability evaluation [14]. In contrast, this study incorporated independent Verification and Validation (V&V) testing conducted by an accredited institution. Hand keypoint recognition accuracy, procedural recognition accuracy, and real-time frame stability (≥60 FPS) satisfied predefined engineering acceptance criteria.

Although the reported 100% recognition accuracy reflects performance under standardized testing conditions, independent validation provides critical evidence of structural robustness and technical reproducibility. Establishing engineering-level reliability is essential when XR is conceptualized as a performance measurement infrastructure rather than solely an experiential learning interface.

### 4.5. Positioning Within State-of-the-Art and Cross-Disciplinary Contexts

Recent developments in medical simulation have increasingly incorporated motion tracking and algorithm-based analytics to differentiate expertise levels and refine procedural assessment [22,23]. In parallel, simulation research has shifted its emphasis from visual realism toward functional fidelity as a determinant of effective skill acquisition [26]. The present study aligns with these developments by demonstrating that continuous sensor-derived performance indicators can be structured into reproducible and incrementally valid assessment metrics within a nursing practicum context.

Rather than focusing solely on immersive experience or subjective learner outcomes, this study operationalizes XR as a measurement-oriented infrastructure grounded in real-time kinematic data and procedural sequencing logic. By empirically establishing structural agreement with expert ratings and incremental explanatory power beyond conventional checklist-based assessments, the findings contribute methodological evidence supporting the integration of sensing architectures into clinical skills evaluation frameworks.

Although the present investigation was conducted within a single nursing education setting, the measurement-oriented design framework may inform future research exploring scalable performance analytics in other professional training contexts. Such extensions, however, require further empirical validation across diverse learner populations and institutional environments.

It should be noted that the usability questionnaire was structured according to ISO 9241-11 domains; however, a fully standardized instrument such as the System Usability Scale (SUS) was not formally implemented. Future research should incorporate validated usability scales to strengthen methodological rigor, enhance reproducibility, and facilitate cross-study comparability.

## 5. Conclusions

This study developed and validated a precision-sensing, controller-free XR-based nursing practicum system and empirically examined the measurement properties of its automated assessment framework. The findings demonstrate that XR-derived performance scores show strong structural agreement with instructor evaluations and provide incremental explanatory value beyond baseline instructor-rated assessments. These results suggest that continuous sensor-based metrics can capture procedural characteristics that may not be fully represented in conventional checklist-based evaluation models.

Independent accredited Verification and Validation testing further supported the system’s technical stability and reproducibility under predefined engineering criteria. Together, these findings indicate that XR environments can be structured to function as data-informed performance measurement platforms in addition to immersive training interfaces.

By integrating standardized nursing protocols with continuous sensing architectures and algorithmic evaluation logic, this study contributes methodological evidence toward enhancing precision and reproducibility in clinical skill assessment.

Several limitations should be acknowledged. The study was conducted within a single institutional context, and accredited V&V testing occurred under controlled environmental conditions. In addition, long-term transfer of XR-derived performance improvements to authentic clinical practice was not directly examined. Future research should investigate multi-site generalizability, performance stability under ecologically variable deployment conditions, and longitudinal clinical skill transfer.

## Figures and Tables

**Table 1:** General Characteristics of Participants (N = 63).

Characteristic	Category	*n*	%
Gender	Male	11	17.5
	Female	52	82.5
Academic Achievement	High	18	28.6
	Medium	35	55.6
	Low	10	15.9
Satisfaction with Nursing Major	High	15	23.8
	Medium	32	50.8
	Low	16	25.4

**Table 2:** Pre–Post Comparison of Instructor-Rated Scores (N = 63).

Variable	Pre-Test	Post-Test	t	*p*
	Mean (SD)	Mean (SD)		
Nursing Knowledge	3.77 (0.61)	4.65 (0.28)	10.624	<0.001
Acquisition	3.39 (0.85)	4.81 (0.35)	11.815	<0.001
Clinical Skills	3.90 (0.50)	4.59 (0.21)	10.961	<0.001
Learning Satisfaction	4.45 (0.46)	4.94 (0.14)	8.370	<0.001

**Table 3:** Differences in Instructor-Rated Learning Gains According to Learner Characteristics (N = 63).

Characteristic (n)		Δ Mean (SD)				t/F	***p***
		Knowledge	Acquisition	Clinical Skills	Satisfaction		
Gender	Male (11)	1.00 (0.81)	0.73 (0.78)	0.71 (0.55)	0.45 (0.47)	−2.791	0.007
	Female (52)	0.86 (0.63)	1.57 (0.93)	0.70 (0.50)	0.51 (0.47)		
Academic Achievement	High (17)	0.74 (0.53)	1.44 (0.83)	0.64 (0.47)	0.46 (0.41)	4.435	0.016
	Medium (36)	0.96 (0.76)	1.21 (0.95)	0.71 (0.54)	0.44 (0.49)		
	Low (10)	0.85 (0.41)	2.17 (0.86)	0.81 (0.49)	0.70 (0.51)		
Major Satisfaction	High (14)	0.75 (0.51)	1.57 (0.82)	0.74 (0.55)	0.55 (0.38)	3.654	0.032
	Medium (33)	0.92 (0.80)	1.14 (0.93)	0.77 (0.52)	0.52 (0.51)		
	Low (16)	0.91 (0.42)	1.87 (0.96)	0.54 (0.44)	0.37 (0.43)		

Δ = Post Instructor − Pre Instructor (5-point scale).

**Table 4:** Hierarchical Regression Analysis Predicting Instructor-Rated Post-Training Performance (N = 63).

Model	Predictor	B	SE	β	t	*p*	R^2^	ΔR^2^
**Step 1**	Instructor Pre Total	0.481	0.082	0.602	5.862	<0.001	0.362	—
**Step 2**	Instructor Pre Total	0.311	0.079	0.389	3.937	<0.001	0.548	0.186
	XR Baseline Score	0.152	0.031	0.452	4.839	<0.001		

**Table 5:** Usability Evaluation Results of the XR Program (N = 63).

Variable	Evaluation Result	Minimum	Maximum	Satisfaction (%)
	Mean (SD)			
Ease of Use	4.52 (0.48)	3.25	5.00	90.4
Interface Intuitiveness	4.61 (0.44)	3.50	5.00	92.2
System Stability	4.58 (0.51)	3.00	5.00	91.6
Immersion	4.67 (0.39)	3.75	5.00	93.4
Overall Usability	4.59 (0.42)	3.38	5.00	91.8

**Table 6:** Technical Stability Issues Related to VR Use (N = 63).

Variable	Number of Occurrences	Incidence Rate (%)
System Error	-	-
VR Sickness	1	1.6
Device Malfunction	1	1.6
Dropout	-	-
Overall Completion	63	100.0

**Table 7:** Verification and Validation Results of Smart Nursing v1.0 Conducted by an Accredited Testing Institution under Controlled Test Conditions.

Variable	Test Result	Acceptance Criteria
User Hand Keypoint Recognition Accuracy	100%	≥80%
Action Recognition Accuracy for XR-Based Nursing Skills Training	100%	≥90%
User Hand Keypoint Detection Speed	≥60 FPS	≥60 FPS

## Data Availability

The data presented in this study are available on request from the corresponding author. The data are not publicly available due to privacy and ethical restrictions.
